# Exploring the impact of external collaboration on firm growth capability: the mediating roles of R&D efforts

**DOI:** 10.1057/s41599-022-01429-5

**Published:** 2022-11-04

**Authors:** Shuting Chen, Dengke Yu

**Affiliations:** School of Public Policy and Administration, Nanchang University, Nanchang, China

**Keywords:** Business and management, Business and management

## Abstract

In today’s business environment with high market turbulence, rapid technological change, and fierce competition, external collaboration and internal efforts in research and development (R&D) become equally important for firm growth. However, little is known about the effects of external collaboration on firm growth that generates along the path from outside to inside. Therefore, this study aims to explore the indirect effects of different types of external collaboration on firm growth capability via R&D efforts. It empirically analyzed a sample of 94 Chinese top-ranking innovative enterprises by applying hierarchical regression and mediation analysis. The results indicate that vertical collaboration, horizontal collaboration, and competitor collaboration are positively and directly related to the firm’s R&D intensity, R&D human capital, and firm growth capability. Furthermore, the firm’s R&D intensity and R&D human capital are positively and directly related to growth capability. The results of mediation analyses showed that R&D intensity mediated the relationship between external collaboration and firm growth capability. However, the results failed to support the mediating role of R&D human capital in the relationship between external collaboration and firm growth capability. This study enriches the literature on open innovation and organizational growth, and provides valuable insights for firm managers and policymakers.

## Introduction

In the past three decades, China has developed from lagging to an emerging economy (Wang et al., 2021). In this process, the rise and development of enterprises play a crucial push-pull role. However, there is a risk of failure at any stage of enterprise development (Josefy et al., 2017). Statistics showed that more than one million enterprises close every year in today’s China. The average life expectancy of Chinese enterprises is only 3.7 years, of which the average life of small and medium-sized enterprises is 2.5 years and that of large enterprises is 7–8 years. More seriously, the business environment characterized by rapid technological change, market volatility, intense competition, and the global COVID-19 pandemic (Zouaghi et al., 2018) still expands the risk over time. Some unanticipated, subversive, and new challenges brought by these external environments continuously threaten the survival of weakly adaptable enterprises (Guo et al., 2021). Therefore, how to maintain the sustainable growth of a firm becomes a noteworthy issue to which global managers are devoted.

Meanwhile, the topic of sustainable organizational growth has received increasing attention in academia. Research from a resource-based view highlighted the importance of internal and external resources in firm growth (Barney and Clark, 2007; Barney et al., 2021), especially internal knowledge possessed by the firm and new knowledge acquired from the outside (Grant, 1996; Martín-de-Castro et al., 2011). Furthermore, scholars who hold the dynamic capability view always believed that firms must ceaselessly develop their internal abilities and explore more external resources to cope with rapidly changing environments to maintain continuous competitive advantage (Eisenhardt and Martin, 2000; Teece, 2007). These studies suggest that a firm’s growth not only relies on its internal impetus but also on external thrust. The internal impetus mainly comes from research and development (R&D) efforts, since R&D activities can create VIRN (valuable, inimitable, rare, and non-substitutable) knowledge and capabilities for firms (Dimitropoulos, 2020; Martínez-Sánchez et al., 2020). In a socio-economic context, a firm’s external thrust often derives from its external links and collaborators. The openness of a firm helps to break organizational boundaries, thus allowing useful knowledge and information from outside to flow within the organization, and sharing resources and risks with partners (Zhu et al., 2019). In short, R&D efforts and external collaboration are considered two enablers of firm growth.

To date, the previous research on the relationship between R&D efforts, external collaboration, and firm growth has mainly focused on the following three streams. First, some studies took R&D effort and external collaboration as two parallel concepts and explored their impacts on firm growth independently (Zouaghi et al., 2018; Garcia Martinez et al., 2019). Second, a few studies investigated the moderation effects of external collaboration on the relationship between R&D efforts and firm growth (Ren et al., 2015; Abdul Basit and Medase, 2019). Third, some other existing empirical studies examined the impact of external collaboration on firm growth considering R&D effort as a moderating variable (Chen et al., 2016; Brinkerink, 2018). The latter two streams are the same in essence. Both of them emphasized and tried to explore the interaction effects. The difference was only reflected in the cognitive bias in the choice of independent and moderating variables. These studies of the three streams provided an overview of the relationships between the three constructs for us from various perspectives. However, the following gaps have yet been addressed: first, most scholars proposed that external collaboration is an enabler of firm growth as a whole, and only a few attempts have investigated the heterogeneous effect of different types of external collaboration; second, existing literature focused on the growth of firm performance, while ignoring that the firm growth capability itself is a multidimensional construct; third, compared to the interaction mechanism between external collaboration and R&D efforts, the outside-to-inside impacting path from external collaboration to R&D efforts lacks due attention; and finally, this topic was not fully discussed in emerging economics.

**Fig. 1:**
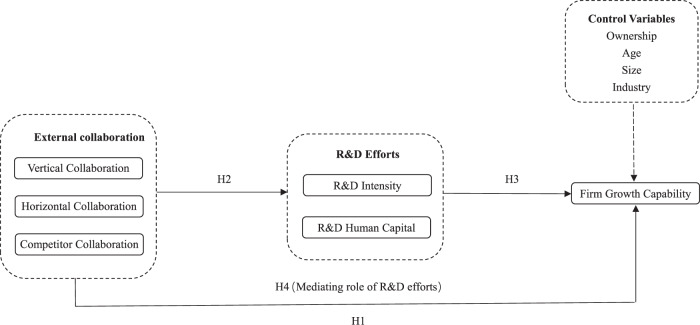
Conceptual framework. The H1, H2, H3, and H4 marked by solid arrows are the hypotheses proposed in the Section “Hypotheses development”. The dotted arrow indicates the effects of control variables which are not highlighted in the hypotheses.

To address the above gaps, this paper aims to explore how different types of external collaboration affect firm growth capability via R&D efforts based on an empirical analysis of the data of 94 top-ranking innovative enterprises in China. In our research framework, we considered three types of external collaboration, i.e., vertical collaboration, horizontal collaboration, and competitor collaboration, and introduced two dimensions of R&D efforts, i.e., R&D intensity and R&D human capital. In the process of data analysis, we firstly examined the direct impact of three types of external collaboration on firm growth capability, and then analyzed the indirect effect via the mediating role of R&D efforts. The conceptual framework is demonstrated in Fig. 1.

The study can make three contributions to the literature. Primarily, it contributes to open innovation theory by linking external collaboration and R&D efforts together. Moreover, it builds a new theoretical framework of firm growth by demonstrating the indirect effect of external collaboration via the mediation of R&D efforts. Third, it provides a context to explore innovation-driven development strategies in innovative firms in emerging economies.

The remainder of this paper is structured as follows. Section “Literature review” presents the literature review of the key constructs of this study. Section “Hypotheses development” develops research hypotheses. Section “Methodology” describes samples, data, measures, and statistical techniques. Section “Data analysis and discussion” outlines our empirical results. Finally, implications, limitations, and future research directions are summarized in Section “Conclusion”.

## Literature review

### External collaboration

Open innovation is a distributed innovation process that depends on consciously managed knowledge flows across boundaries, applying pecuniary and nonpecuniary mechanisms in line with the firm’s business model to guide and motivate knowledge sharing (Chesbrough, 2017). External collaboration is an important construct in open innovation theory. It describes an interdependent and win-win relationship between a firm and its partners linked by interactive, open, and direct communication, which supports the firm’s innovation and experimentation, and thus creates beneficial outcomes for all participants (Jap, 2001). A firm must acquire diverse new knowledge, organize value-creation activities and improve competitive advantage, because in the networked society the locus of innovation resides not inside the firm, but in the interstices between the firm and its external partners (Powell et al., 1996; Wang et al., 2015). Therefore, a firm needs to constantly develop external collaboration and knowledge alliances to meet new business challenges and achieve sustainable development.

It is most popular and widespread to study external collaboration from the perspective of its breadth and depth (Laursen and Salter, 2006; Ferreras-Méndez et al., 2015; Zhu et al., 2019). Collaboration breadth reflects the number of external partners that a firm relies upon in its innovative activities, and collaboration depth reflects the intensity of collaboration with each type of partner. The two jointly represent the firm’s openness to the external environment, with a focus on knowledge sharing with different collaborative objects, such as suppliers, customers, competitors, governments, universities, etc. (Dong and Netten, 2017). This research stream bundles different types of external partners together, regardless of the heterogeneity among them (Chen et al., 2016). However, managers do not randomly choose partners to broaden and deepen their corporate collaboration strategies, since various partners do have different impacts on a firm. In consequence, it is necessary to classify external collaboration from a specialization perspective and explore the impacts of different types of external partners on firm development.

After realizing the deficiency, scholars began to develop the construct from other perspectives. For example, Faems et al. (2005) categorized external collaboration into exploitation-oriented collaboration (i.e. collaboration with customers and suppliers) and exploration-oriented collaboration (i.e. collaboration with universities and research institutions). Sofka and Grimpe (2010) divided external collaboration into three subdimensions, including the science-driven dimension (i.e. collaboration with universities and public research centers), market-driven dimension (i.e. communication with customers and competitors), and supply driven dimension (i.e. exchange with suppliers and obtain information from conferences and trade fairs).

By integrating these views and aligning with past studies (Stefan and Bengtsson, 2017; Garcia Martinez et al., 2019), we classified external collaboration into vertical collaboration, horizontal collaboration, and competitor collaboration from a more holistic perspective. Specifically, vertical collaboration refers to the information transfer and knowledge interaction between a firm and its suppliers and customers along the industrial chain; horizontal collaboration refers to the knowledge exchange of a firm with its stakeholders in the social environment, such as government organizations, universities and educational institutions, consultancy firms, venture capitalists, and trade fairs and exhibition; and finally, competitor collaboration is a special horizontal collaboration which combines collaboration and competition when the firm treats the relationship with its competitors.

### R&D efforts

R&D efforts refer to the degree of efforts made by a firm in R&D investment and activities (Molina-Morales and Expósito-Langa, 2012). R&D efforts reflect the recruitment of excellent R&D personnel, the investment of R&D funds, the purchase of advanced R&D equipment, and the expansion of various R&D actions. The return rate of R&D investment tends to be high. It is usually in 20–30% range, and sometimes it may be as high as 75% or so (Chen, 2018). This stunning rate of return has encouraged thousands of firms across the globe to make more efforts in R&D activities. Additionally, R&D can help a firm to develop new products, build knowledge bases, enhance development capabilities and maintain a competitive advantage (Martínez-Sánchez et al., 2020). Therefore, though many firms have met with success through the development of external resources on the basis of broad cooperation, other firms still adhere to innovation-driven development strategies and continuously invest into R&D activities heavily.

Owing to the complexity and diversity of R&D efforts, scholars classified it from different angles. For instance, Lee and Wu (2016) categorized R&D efforts into R&D expenses (i.e. current R&D expenditures) and R&D capital (i.e. perpetual inventory of the past R&D investment). Son and Zo (2021) divided R&D efforts into three aspects according to the type of invested resources, namely technological dimension, human dimension, and financial dimension. Following Zouaghi et al. (2018) and Garcia Martinez et al. (2019), in this study, we argued that R&D effort is composed of R&D intensity and R&D human capital. The former is recognized as the degree to which a firm invests funds in the R&D aspect, and the latter is identified as a capability created by the knowledge experience, and skills of R&D personnel (Ployhart and Moliterno, 2011). Both are indispensable drivers for a firm to maintain sustainable growth.

### Firm growth capability

Firm growth is a process in which the organization and function of the firm system are constantly differentiated, thus promoting the firm’s continuous expansion, continuous adaptation to the environment, and benign interaction with the environment (Penrose, 1959). In other words, firm growth refers to a process of development and evolution through quantitative and qualitative growth under the premise of the continuous existence of the firm (Coad and Guenther, 2014). Quantitative growth is reflected in the firm’s surface, including performance improvement, market share expansion, profit raising, etc., while qualitative growth represents the firm’s essential changes, such as learning capability enhancement, organizational structure optimization, business model innovation, and competitiveness improvement.

Two research streams on firm growth have been differentiated by literature, i.e., endogenous and exogenous firm growth theory (Audretsch et al., 2014). The former focuses on the endogenous factors that drive firm growth, such as in-house resources, organizational systems, and innovation capabilities. On the contrary, the latter focuses on the exogenous factors that drive firm growth, such as market opportunity, environmental uncertainty, public policy, and social resources. These two research streams are equally important, and it makes sense to take into account the synergy between them in a framework.

Scholars have attached great importance to the measurement of firm growth capability (Erhardt, 2021). From a macro perspective, Dalgıç and Fazlıoğlu (2021) emphasized that firm growth capability can be assessed from employment growth and sales growth. From a micro perspective, Yu and Yan (2021) argued that firm growth capability should be evaluated by income growth and assets growth. However, these studies generally measured “how much” a firm grows, but ignored investigating “how” the firm grows (McKelvie and Wiklund, 2010). To cover this shortage, we explored firm growth capability from both financial and process dimensions. Among them, the financial dimension reflects the improvement of the firm’s financial capability, which is measured by the variances including debt-paying ability, operating ability, profitability, and development ability. The process dimension reflects the enhancement of the firm’s capability of a whole business process during its growth, which covers R&D ability, manufacturing ability, marketing ability, and serviceability.

## Hypotheses development

### External collaboration and firm growth capability

Today’s fast-paced business environment requires firms to develop external relational capital as external collaboration with a variety of partners can bring many benefits to sustainable growth. First, vertical collaboration enhances the value-added capability of firms in the context of the value chain. Cooperation with suppliers can help firms to complement their technological bases, improve product development efficiency, and save logistics costs (Un et al., 2010). Cooperation with customers enables firms to get market information and respond to new demands rapidly, thereby reducing risks associated with market changes and new product development (Rodriguez et al., 2017). Since these partners are in the same industrial chain and share innovation benefits, they are willing to jointly contribute to better innovation performance (Chen et al., 2016). Moreover, the firms that build horizontal alliances with stakeholders in a macro environment are more likely to be strategically motivated to maintain long-term development. For instance, collaboration with governments can provide firms with policy and financial subsidies, which often specifically fund firms for new product development or technical improvement. Alliances with universities and educational institutions can provide firms with tailored and cutting-edge technologies and knowledge (Tsai and Hsieh, 2009). Consultants can help firms improve their development strategies and organizational systems. Venture capital firms not only provide direct funds but also bring valuable market information and technical expertise (Chen et al., 2016). Trade fairs and exhibitions are important platforms for firms to communicate with external institutions in the field of new technologies, products, and talent exchange (Sofka and Grimpe, 2010). In addition, competitors may collaborate to achieve the technological breakthrough, develop industry standards, share resources or channels, and intercept potential comers (Tether, 2002). Competitor collaboration can enhance firms’ strategic capabilities and improve their competitive position in the industrial ecosystems to a certain extent (Chen et al., 2016).

Several studies have provided empirical evidence for the positive impact of external collaboration on firm growth capability. For example, Wang et al. (2015) reported that external effective collaboration significantly enhances market performance. Chen et al. (2016) suggested that different types of external knowledge sourcing contribute to the improvement of firms’ innovative performance. Zouaghi et al. (2018) found that firms with broad open collaboration strategies tended to have higher innovation performance. Thus, we propose the following hypothesis:

*H1: (a) Vertical collaboration, (*b) *horizontal collaboration, and (c) competitor collaboration have positive impacts on firm growth capability*.

### External collaboration and R&D effort

We argued that external collaboration stimulates the growth of R&D efforts based on the push and pull theory. From a pushing perspective, firms would feel the pressure when their collaborators expect long-term behaviors involving R&D efforts (Molina-Morales and Expósito-Langa, 2012). Many collaborations are implemented based on R&D strategies (Huang et al., 2015). From a pulling perspective, firms are self-motivated by the R&D performance which is sharply strengthened by external collaboration. Firms gain a wealth of valuable, up-to-date, and heterogeneous knowledge and information through external cooperation, broadening their horizons, inspiring them in R&D activities, and enabling them to bridge the technological gaps (Zhu et al., 2019). Under the double effects of pull and push, external collaboration stimulates firms to carry out more targeted R&D efforts on collaborative innovation, which is also beneficial for firms to avoid obsolescence and maintain a sustainable competitive advantage. Concretely, firms will invest more R&D to develop new products recommended by leading customers. Firms will conduct more R&D activities to acquire their competitors’ knowledge and imitate their advanced products. In addition, meetings on cutting-edge technologies and market opportunities from universities and research institutions will facilitate firms to invest in R&D strategies based on industry-academia cooperation.

Several studies have addressed the positive relationship between external collaboration and R&D efforts. Garcia Martinez et al. (2017) argued that firms with high alliance portfolio diversity are more willing to invest in R&D capability. Zhu et al. (2019) found that multiple information from external partners can serve as an impetus to innovative actions, thus facilitating the implementation of R&D activities. Vlaisavljevic et al. (2021) proposed that the increased proactiveness in forming technical alliances can improve the investment in R&D human capital. Consequently, we propose the following hypothesis:

*H2: (a) Vertical collaboration, (*b) *horizontal collaboration, and (c) competitor collaboration have positive impacts on R&D efforts*.

### R&D efforts and firm growth capability

With the advance of a knowledge-based economy, R&D efforts become more and more essential for firms. R&D efforts, which reflect R&D intensity and R&D human capital from an investment perspective, play an important role in the implementation of a firm’s innovation-driven development strategy. On the one hand, R&D intensity is often considered a contributor to absorptive capacity, which is an essential “ability to recognize the value of new information, assimilate it, and apply it to commercial ends” (Cohen and Levinthal, 1990; Zahra and George, 2002). In addition, R&D intensity is also a key factor in determining a firm’s capability to innovate (Brinkerink, 2018). On the other hand, R&D human capital composed of knowledgeable, creative, and skilled R&D employees is the most important resource for a firm’s innovative development (Gupta et al., 2020). Resource-based theorists have always highlighted that R&D human capital is an important source of core competitiveness and sustainable competitive advantage because the tacit knowledge mastered by R&D employees has VIRN characteristics (Coff, 1997). In firms, R&D employees are responsible for translating their creative tacit knowledge into new products and economic performance (Delgado-Verde et al., 2016).

Prior studies have demonstrated the proposition that R&D efforts positively impact firm growth capability. Li et al. (2010) suggested that R&D intensity has long been recognized as an important driver for the survival of firms in the high-tech software industry. Kim and Lee (2016) reported that R&D intensity is a vital firm-specific dynamic capability that significantly affects firm development. Siepel et al. (2017) demonstrated that general and specific human capital in the workforce plays a crucial role in shaping the long-run growth of high-tech ventures. Garcia Martinez et al. (2019) highlighted that firms with high levels of R&D human capital are better able to survive and thrive in uncertain financial conditions. Therefore, we propose the hypothesis as follow.

*H3: R&D effort has a positive impact on firm growth capability*.

### The mediating role of R&D effort

The above analysis does have indicated us to develop a mediation model. In this section, we made more efforts to demonstrate the mediating role of R&D efforts in the relationship between external collaboration and firm growth capability by employing incentive theory and dialectical logic. Dialectical logic shows that internal factors are the primary causes of the development of things, followed by external factors. As the incentive theory states, an organism’s behavior is motivated by both internal and external factors, but the effects of external factors need to be mediated by internal factors. In the context of this study, for the target of firm growth, external collaboration is the external factor and R&D efforts are the internal factors, and the relationship between them should follow the logic of incentive theory.

The specific affecting mechanism is as follows. First, in the modern enterprise system, R&D is a necessary function of firm growth (Abdul Basit and Medase, 2019). Firms can create unique products to differentiate themselves from competitors, thereby obtaining competitiveness through R&D efforts. The role of external collaboration in this process is to guide the enterprise to develop products suitable for the market and customers. Second, to better satisfy stakeholders in the external environment from a long-term perspective, firms are willing to do more R&D efforts based on external collaboration, since the latter enables them to obtain more heterogeneous resources and knowledge that are beneficial to the improvement of R&D innovation capability (Zhu et al., 2019). Hence, to a certain extent, the external collaboration will affect the firm’s decision-making on the direction, scale, and structure of R&D investment, thereby affecting the firm’s sustainable growth. Based on these considerations, we propose the following hypothesis.

*H4: R&D effort mediates the relationship between* (a) v*ertical collaboration, (*b*) horizontal collaboration, and (c) competitor collaboration and firm growth capability*.

## Methodology

### Sample

Strategy& and PwC released the 2018 Global Innovation 1000 Study, reporting the world’s top-ranking 1000 listed companies with the highest R&D expenditure. Together, they accounted for 40% of total global R&D expenditure. This study shows that R&D expenditures increase worldwide, but most notably in China, rising 34.4 percent in the year. As an emerging country with rapid development in recent years, China has made great contributions to the development of the global new economy.

We collected and analyzed data of Chinese innovative enterprises ranked in the above study for three reasons. First, these enterprises are in line with the sustainable development strategy advocated by the Chinese government. Most of them are leaders in their respective industries. Second, these enterprises attach great importance to R&D and have made great efforts in R&D. Third, under the support of sufficient resources and attraction of sustainable development, most of them have implemented external collaboration and developed cooperative alliances. Moreover, the information disclosure system involving listed companies provides us with more convenience for data collection.

We followed the following procedures to select our sample. First, we picked out 175 Chinese companies in the 2018 Global Innovation 1000 Study. Second, 76 Chinese companies listed in Hong Kong, Taiwan, and United States were excluded because of the difficulty of data collection. Third, even another 5 companies in mainland China with missing data were also excluded. Finally, 94 Chinese innovative companies listed on Shenzhen and Shanghai Stock Exchanges (A share) were selected as our sample for data analysis.

Table 1 displays the characteristics of our sample. The mainly involving industries included the information technology industry (26.6%), capital goods industry (28.7%), advanced materials industry (17.0%), automobiles and components industry (11.7%), consumer durables and apparel industry (7.4%), and retailing and media industry (2.1%), healthcare industry (4.3%) and energy industry (2.1%). The companies comprised 60.6% state-owned firms and 39.4% non-state-owned firms. Among them, 17.0% hired less than 10000 employees, 42.6% between 10001 and 30000, 19.1% between 30001 and 50000, and the other 21.3% hired more than 50000 employees. In addition, the sample aged 6–10 years accounted for 7.4%, 11–15 years for 12.8%, 16–20 years for 40.4%, 21–25 years for 31.9%, and finally more than 25 years for 7.4%.

**Table 1:** Sample characteristics.

Item	Category	Number	Percentage (%)
*Age*	6–10 years	7	7.4
	11–15 years	12	12.8
	16–20 years	38	40.4
	21–25 years	30	31.9
	>25 years	7	7.4
*Size* (employee number)	1–10000	16	17.0
	10001–30000	40	42.6
	30001–50000	18	19.1
	>50000	20	21.3
*Ownership*	State-owned	57	60.6
	Non-state-owned	37	39.4
*Industry*	Information technology	25	26.6
	Capital Goods	27	28.7
	Advanced materials	16	17.0
	Automobiles and components	11	11.7
	Consumer durables and apparel	7	7.4
	Retailing and media	2	2.1
	Healthcare	4	4.3
	Energy	2	2.1

### Data

The data collection proceeded in two stages. In the first stage, we built composite scales for measuring external collaboration and process dimensions of firm growth capability. We marked the scales based on a content analysis of the company’s disclosed information. In the second stage, the data of other variables were collected from China Stock Market & Accounting Research Database (CSMAR Database).

Following the increasing use of panelists in research (e.g., Zott and Amit, 2007), we set up a panel composed of 3 members, including 1 professor and 2 doctoral students. First, the professor carefully selected our panelists from his research team, requiring that the selected team members should have a good understanding of the firm’s external collaboration and growth capability. After choosing the most qualified candidates, the professor claimed the two selected members (doctoral students) to carefully read the information, announcements, and documents of total sample companies, get familiar with the details of external collaboration and growth capability, and develop measurement scales by following inductive logic. Next, the professor made further training for the two doctoral students, who were authorized as expert raters in data collection and analysis. In addition, the raters were provided with written guidelines on the proper way to address survey items. The underlying materials for data collection include annual financial reports, corporate social responsibility reports, investment analysts’ reports, company news, company websites, and other company announcements between 2016 and 2020. It took every rater about six months from October 2020 to April 2021 to collect data on external collaboration and firm growth capability’s process dimension. To reduce the influence of common method bias, the process of data collection was divided into two stages: the one is to collect the data of independent and control variables, and the other is to collect the data of mediating and dependent variables. The interval between the two is one month. The lack of readily available data obliged us to draw on primary sources of data and constructed a unique, manually collected dataset. The method also prevented us from collecting time-series data. Finally, we evaluated the consistency by conducting a pairwise comparison of two raters’ scores, yielding a Pearson correlation coefficient of 0.951 (*p* < 0.01). For the different scores, the two raters discussed with each other under the guidance of the professor and finally reached a consensus.

The data of other variables were drawn from CSMAR Database, which is a research-oriented accurate database in the economic and financial field compiled by Shenzhen CSMAR Data Technology Co., Ltd. The database combines the actual national conditions of China and draws on the professional standards of authoritative databases such as CRSP, COMPUSTAT, TAQ, and THOMSON. The period of data collection was fixed as the year 2018. To control the influence of extreme values on research results, the data collected from CSMAR were winsorized.

### Measures

#### Independent variables

In line with previous studies (Garcia Martinez et al., 2019), we distinguished three types of external collaboration and set them as independent variables. That is vertical, horizontal, and competitor collaboration. Among them, vertical collaboration (*VC*) was measured by the total score of collaboration with suppliers and customers; horizontal collaboration (*HC*) was measured by the total score of collaboration with governments, universities and educational institutions, consultancy firms, venture capital investment firms, trade fairs and exhibitions, and others; and competitor collaboration (*CC*) was measured by the score of collaboration with competitors. The score of collaboration with each type of collaborator was coded as a binary variable which was measured by 1 and 0. Among them, 1 represents that collaboration widely and deeply happens and 0 represents the reverse side.

#### Dependent variables

Considering several studies have explored the relationship between external collaboration and innovation performance (Findik and Beyhan, 2015; Lu and Yu, 2020), we need to define and measure firm growth capability (*FGC*), the dependent variable of our study, from a new perspective that differs from the prior studies. We realized that firm growth capability is a much more comprehensive construct than innovation performance, and so we tried to measure it from both financial and process dimensions according to the theory of balanced scorecard. We carried out a data pre-processing for the financial and process ones into [0,1] interval and measured the firm growth capability by the mean value of their scores. First, the financial dimension of firm growth capability was measured by four indexes, i.e., debt-paying ability, operating ability, profit ability and development ability. Among them, the debt-paying ability was assessed by the current ratio (current assets/current liabilities); operating ability was evaluated by inventory turnover (operating costs/average inventory); profitability was measured by operating profit ratio (operating profit/operating income); and development ability was evaluated by the operating income growth rate (current year’s operating income/last year’s operating income −1). Through principal component analysis, we calculated the weighted score of the financial dimension. Second, we independently developed a scale for measuring the process dimension of firm growth capability. Four items, i.e. “the enterprise has established a global R&D center and created a global intelligent R&D platform”, “the enterprise has advanced intelligent manufacturing system”, “the enterprise has diversified, advanced, and intelligent sales system for offline and online sales”, and “the enterprise has advanced, convenient and intelligent service system for dealing with pre-sale, sale and after-sale issues”, were developed to respectively measure research and development ability, manufacturing ability, marketing ability, and serviceability. Obviously, the firm growth capability measured in our study includes but also goes beyond the connotation of innovation performance that was concerned by predecessors (Findik and Beyhan, 2015; Lu and Yu, 2020). Considering the difficulty to obtain an objective score for the measures, we deemed the use of perceptual coding of our raters. The involved items were quantified on a five-point scale. After coding, the scores of total items were aggregated and averaged as the final score of the process dimension.

#### Mediating variables

R&D intensity and R&D human capital constitute R&D effort. Among them, R&D intensity (*RI*) was measured by the ratio of a firm’s R&D expenditure to operating income (Kim and Lee, 2016), and R&D human capital (*RHC*) was measured by the percentage of highly skilled R&D workers (researchers and technicians) (Teixeira and Tavares-Lehmann, 2014).

#### Control variables

Learning from the previous study (Yu and Yan, 2021), we designed four control variables as the alternative explanations for firm growth. First, we set *ownership* as a dummy variable that controls for potential variations between state-owned enterprises (coded as 1) and private-owned, foreign-owned, or other types of enterprises (coded as 0). Second, the *age* was assessed by subtracting the year of firm establishment from the year in which the survey was conducted. Third, the *size* was measured by the natural logarithm of the employee scale. Finally, the *industry* as a dummy variable was coded as 1 when a firm belongs to the industry of advanced materials, consumer discretionary, and healthcare and energy. Otherwise, it was coded as 0.

### Statistical technique

Following the suggestion of Hox (1994), we tested direct effects by hierarchical regression analysis, using SPSS 24 software. Following the recommendation of Preacher and Hayes (2008), We tested mediating effects by bias-corrected bootstrapping procedure, using PROCESS v. 3.3.

### Model development

Drawing on hierarchical regression analysis, we constructed the following models to test our hypotheses.

First, to measure the impacts of external collaboration on firm growth capability that were proposed by H1(a, b, c), we constructed the models M1-M5. Among them, model M1 tests the effect of control variables, models M2-M4 respectively test the effects of vertical collaboration, horizontal collaboration, and competitor collaboration, and model M5 tests their combined effect.

1 $${FGC_i = a_0 + a_1Controls_i + \mu }_i$$

2 $${FGC_i = b_0 + b_1VC_i + b_2Controls_i + \mu }_i$$

3 $$\begin{array}{*{20}{c}} {FGC_i = c_0 + c_1HC_i + c_2Controls_i + \mu _i} \end{array}$$

4 $$\begin{array}{*{20}{c}} {FGC_i = d_0 + d_1CC_i + d_2Controls_i + \mu _i} \end{array}$$

5 $$\begin{array}{*{20}{c}} {FGC_i = e_0 + e_1VC_i + e_2HC_i + e_3CC_i + e_4Controls_i + \mu _i} \end{array}$$

Second, we developed the models M6-M15 to test the effects of external collaboration on R&D efforts that were proposed by H2(a, b, c). Among them, M6 and M11 were set for measuring the effects of control variables on *RI* and *RHC* respectively; the models M7-M9 measure the independent effects of *VC*, *HC*, and *CC* on *RI*, and similarly the models M12-M14 measure their independent effects on *RHC*; and the models M10 and M15 test their combined effects on *RI* and *RHC* respectively.

6 $$\begin{array}{*{20}{c}} {RI_i = f_0 + f_1Controls_i + \mu _i} \end{array}$$

7 $$\begin{array}{*{20}{c}} {RI_i = g_0 + g_1VC_i + g_2Controls_i + \mu _i} \end{array}$$

8 $$\begin{array}{*{20}{c}} {RI_i = h_0 + h_1HC_i + h_2Controls_i + \mu _i} \end{array}$$

9 $$\begin{array}{*{20}{c}} {RI_i = j_0 + j_1CC_i + j_2Controls_i + \mu _i} \end{array}$$

10 $$\begin{array}{*{20}{c}} {RI_i = k_0 + k_1VC_i + k_2HC_i + k_3CC_i + k_4Controls_i + \mu _i} \end{array}$$

11 $$\begin{array}{*{20}{c}} {RHC_i = l_0 + l_1Controls_i + \mu _i} \end{array}$$

12 $${RHC_i = m_0 + m_1VC_i + m_2Controls_i + \mu _i}$$

13 $$\begin{array}{*{20}{c}} {RHC_i = n_0 + n_1HC_i + n_2Controls_i + \mu _i} \end{array}$$

14 $$\begin{array}{*{20}{c}} {RHC_i = p_0 + p_1CC_i + p_2Controls_i + \mu _i} \end{array}$$

15 $$\begin{array}{*{20}{c}} {RHC_i = q_0 + q_1VC_i + q_2HC_i + q_3CC_i + q_4Controls_i + \mu _i} \end{array}$$

Next, we built the models M16-M18 to examine the impacts of R&D effort on firm growth capability that were proposed by H3. Among them, the models M16 and M17 measured the independent effects of *RI* and *RHC* respectively, and in M18 we measured their combined effect.

16 $$\begin{array}{*{20}{c}} {FGC_i = r_0 + r_1RI_i + r_2Controls_i + \mu _i} \end{array}$$

17 $$\begin{array}{*{20}{c}} {FGC_i = s_0 + s_1RHC_i + s_2Controls_i + \mu _i} \end{array}$$

18 $$\begin{array}{*{20}{c}} {FGC_i = t_0 + t_1RI_i + t_2RHC_i + t_3Controls_i + \mu _i} \end{array}$$

Finally, we developed the models M19-M21 to measure the mediating effects of *RI* and *RHC* on the relationship between external collaboration (*VC*, *HC*, and *CC* respectively) and *FGC* that were proposed by H4(a, b, c).

19 $${FGC_i = v_0 + v_1VC_i + v_2RI_i + v_3RHC_i + v_4Controls_i + \mu }_i$$

20 $$\begin{array}{*{20}{c}} {FGC_i = w_0 + w_1HC_i + w_2RI_i + w_3RHC_i + w_4Controls_i + \mu _i} \end{array}$$

21 $${FGC_i = x_0 + x_1CC_i + x_2RI_i + x_3RHC_i + x_4Controls_i + \mu }_i$$

where *Controls* indicate control variables, including ownership, age, size (Ln), and industry, *μ* indicates the random disturbance, and *i* denotes the number of samples.

## Data analysis and discussion

### Descriptive statistics and correlations

Table 2 shows the mean, standard deviation, and correlation coefficient of the main variables. We found that R&D intensity is significantly and positively correlated with horizontal collaboration *(r* = *0.547, p* < *0.01)* and competitor collaboration *(r* = *0.176, p* < *0.1)*, but not significantly correlated with vertical collaboration *(r* = *0.054, p* > *0.1)*. R&D human capital is significantly and positively correlated with horizontal collaboration *(r* = *0.585, p* < *0.01)*, but not significantly correlated with vertical collaboration *(r* = *0.065, p* > *0.1)* and competitor collaboration *(r* = *0.078, p* > *0.1)*. Firm growth capability is significantly and positively correlated with horizontal collaboration *(r* = *0.545, p* < *0.01)*, competitor collaboration *(r* = *0.178, p* < *0.1)*, R&D intensity *(r* = *0.685, p* < *0.01)* and R&D human capital *(r* = *0.597, p* < *0.01)*, but is also not significantly correlated with vertical collaboration *(r* = *0.134, p* > *0.1)*. In a word, positive correlation relationships do exist between the core variables involved in this study, since most of the correlations are significant. This provides some preliminary evidence for a part of our research hypotheses.

**Table 2:** Descriptive statistics and correlations.

Variables	Mean	S.D.	1	2	3	4	5	6	7	8	9	10
1 *Ownership*	0.610	0.491	1									
2 *Age*	18.910	5.015	−0.066	1								
3 *Size (Ln)*	10.149	1.079	0.052	−0.225^**^	1							
4 *Industry*	0.550	0.500	−0.111	−0.328^***^	−0.109	1						
5 *RI*	0.063	0.053	−0.215^**^	0.024	−0.471^***^	0.253^**^	1					
6 *RHC*	0.195	0.166	−0.090	0.015	−0.457^***^	0.358^***^	0.742^***^	1				
7 *VC*	0.480	0.563	0.067	−0.027	0.182*	−0.034	0.054	0.065	1			
8 *HC*	1.660	1.372	−0.137	0.155	−0.334^***^	0.089	0.547^***^	0.585^***^	0.199^*^	1		
9 *CC*	0.380	0.489	−0.082	0.088	0.019	−0.260^**^	0.176^*^	0.078	0.069	0.132	1	
10 *FGC*	0.460	0.169	−0.390^***^	0.132	−0.348^***^	0.190^*^	0.685^***^	0.597^***^	0.134	0.545^***^	0.178^*^	1

*N* = 94. *p < 0.1, **p < 0.05, ***p < 0.01 (two-tailed tests).

In addition, the highest correlation value is 0.742, below the threshold value of 0.75 (Tsui et al., 1995), suggesting no serious multicollinearity problem within the variables. It is confirmed again by calculating variance inflation factor (VIF) values. The highest VIF value in all models is 2.523, well below the rule of thumb cut-off of 10, indicating that the multicollinearity would not significantly influence our research results (O’Brien, 2007).

### Hypotheses tests

#### Direct effects

Through hierarchical regression analysis, the effects of external collaboration on firm growth capability are shown in Table 3. The results of M1-M5 models show that vertical collaboration *(β* = *0.403, p* < *0.05)*, horizontal collaboration *(β* = *0.314, p* < *0.01)* and competitor collaboration *(β* = *0.413, p* < *0.05)* have significantly positive effects on firm growth capability. Hence, hypothesis H1 is supported. However, when they work together on firm growth capability, the impact of horizontal collaboration *(β* = *0.272, p* < *0.01)* is still significantly positive, but the impacts of vertical collaboration *(β* = *0.203, p* > *0.1)* and competitor collaboration *(β* = *0.278, p* > *0.1)* become insignificant. It indicates that the impact of horizontal collaboration on firm growth capability is much stronger than the impacts of vertical and competitor collaborations, and would cover up their effects.

**Table 3:** The effects of external collaboration on firm growth capability.

	Dependent variable: *FGC*				
	M1	M2	M3	M4	M5
Constant	0.270	0.093	−0.268	0.022	−0.453^**^
*Control Variables*					
*Ownership*	−0.718^***^	−0.744^***^	−0.625^***^	−0.671^***^	−0.620^***^
*Age*	0.092	0.088	0.045	0.095	0.051
*Size (Ln)*	−0.293^***^	−0.334^***^	−0.166^*^	−0.291^***^	−0.202^**^
*Industry*	0.299	0.300	0.229	0.411^**^	0.314^*^
*Independent Variables*					
*VC*		0.403^**^			0.203
*HC*			0.314 ^***^		0.272^***^
*CC*				0.413^**^	0.278
*Goodness-of-fit*					
R^2^	0.280	0.329	0.440	0.317	0.469
Adj R^2^	0.248	0.291	0.408	0.278	0.425
F	8.648^***^	8.646^***^	13.821^***^	8.179^***^	10.838^***^
Maximum VIF	1.228	1.228	1.241	1.273	1.293
Durbin-Wastson	1.931	2.009	2.109	1.946	2.192

*N* = 94. *p < 0.1, **p < 0.05, ***p < 0.01 (two-tailed tests).

The effects of external collaboration on R&D effort are shown in Table 4. According to the results of the M6-M10 models, vertical collaboration *(β* = *0.283, p* < *0.1)*, horizontal collaboration *(β* = *0.306, p* < *0.01)*, and competitor collaboration *(β* = *0.486, p* < *0.05)* have significantly positive effects on R&D intensity. When they work together, the impacts of horizontal collaboration *(β* = *0.276, p* < *0.01)* and competitor collaboration *(β* = *0.361, p* < *0.05)* are still significant, but the impact of vertical collaboration becomes insignificant *(β* = *0.075, p* > *0.1)*. It reveals the importance of horizontal and competitor collaborations to the inducement of R&D financial investment. According to the results of M11-M15 models, vertical collaboration *(β* = *0.285, p* < *0.1)*, horizontal collaboration *(β* = *0.347, p* < *0.01)* and competitor collaboration *(β* = *0.363, p* < *0.1)* have significantly positive effects on R&D human capital. When they work together, the impact of horizontal collaboration *(β* = *0.327, p* < *0.01)* is still significantly positive, but the impacts of vertical collaboration *(β* = *0.051, p* > *0.1)* and competitor collaboration *(β* = *0.218, p* > *0.1)* become insignificant. It demonstrates the relatively stronger impact of horizontal collaboration. In any case, the results show that hypothesis H2 is accepted.

**Table 4:** The effects of external collaboration on R&D efforts.

	Dependent variable: R&D Efforts									
	*RI*					*RHC*				
	M6	M7	M8	M9	M10	M11	M12	M13	M14	M15
Constant	0.023	−0.101	−0.502^**^	−0.269	−0.700^***^	−0.311^*^	−0.437^**^	−0.906^***^	−0.530^**^	−1.027^***^
*Control Variables*										
*Ownership*	−0.356^*^	−0.374^**^	−0.265	−0.301^*^	−0.238	−0.064	−0.083	0.038	−0.023	0.054
*Age*	−0.032	−0.034	−0.077	−0.029	−0.071	0.023	0.020	−0.029	0.025	−0.025
*Size (Ln)*	−0.450^***^	−0.479^***^	−0.326^***^	−0.448^***^	−0.344^***^	−0.416^***^	−0.445^***^	−0.276^***^	−0.414^***^	−0.288^***^
*Industry*	0.348^*^	0.348^*^	0.280	0.480^**^	0.385^**^	0.633^***^	0.634^***^	0.556^***^	0.732^***^	0.619^***^
*Independent Variables*										
*VC*		0.283^*^			0.075		0.285^*^			0.051
*HC*			0.306^***^		0.276^***^			0.347^***^		0.327^***^
*CC*				0.486^**^	0.361^**^				0.363^*^	0.218
*Goodness-of-fit*										
R^2^	0.293	0.317	0.445	0.344	0.475	0.306	0.331	0.502	0.335	0.513
Adj R^2^	0.261	0.278	0.413	0.307	0.432	0.275	0.293	0.473	0.298	0.473
F	9.200^***^	8.165***	14.104^***^	9.247^***^	11.105^***^	9.832^***^	8.718^***^	17.717^***^	8.882^***^	12.926^***^
Maximum VIF	1.228	1.228	1.241	1.273	1.293	1.228	1.228	1.241	1.273	1.293
Durbin-Wastson	1.814	1.816	1.946	1.750	1.920	1.738	1.723	1.721	1.679	1.698

*N* = 94. *p < 0.1, **p < 0.05, ***p < 0.01 (two-tailed tests).

The effects of R&D effort on firm growth capability are depicted in Table 5. The results of M16 and M17 show that both R&D intensity *(β* = *0.610, p* < *0.01)* and R&D human capital *(β* = *0.543, p* < *0.01)* have significantly positive effects on firm growth capability. The results of model M18 show that R&D intensity *(β* = *0.452, p* < *0.01)* and R&D human capital *(β* = *0.245, p* < *0.05)* have concurrently significant and positive impacts on firm growth capability. Hence, hypothesis H3 is accepted.

**Table 5:** The effects of R&D efforts on firm growth capability.

	Dependent variable: *FGC*			
	M1	M16	M17	M18
Constant	0.270	−0.256^*^	0.439^***^	0.336^**^
*Control Variables*				
*Ownership*	−0.718^***^	−0.501^***^	−0.683^***^	−0.541^***^
*Age*	0.092	0.111	0.080	0.101
*Size (Ln)*	−0.293^***^	−0.018	−0.067	0.012
*Industry*	0.299	0.086	−0.045	−0.013
*Independent Variables*				
*RI*		0.610^***^		0.452^***^
*RHC*			0.543^***^	0.245^**^
*Goodness-of-fit*				
R^2^	0.280	0.543	0.485	0.567
Adj R^2^	0.248	0.517	0.455	0.537
F	8.648^***^	20.934^***^	16.547^***^	18.988^***^
Maximum VIF	1.228	1.413	1.442	2.523
Durbin-Wastson	1.931	2.058	2.051	2.075

*N* = 94. *p < 0.1, **p < 0.05, ***p < 0.01 (two-tailed tests).

#### Indirect effects

We used the bias-corrected bootstrapping method to test the indirect effects. The mediation analysis was performed at a 95% confidence interval based on 10,000 bootstrap samples. The confidence interval of the lower and upper limits was computed to check whether the indirect effects are significant. If zero is not contained in the confidence interval, it indicates that the indirect effect is significant; otherwise, the indirect effect is insignificant. The results are shown in Table 6. We found that the indirect effects of vertical collaboration *(estimate* = *0.123, 95% CI* = *[0.001, 0.305])*, horizontal collaboration *(estimate* = *0.126, 95%CI* = *[0.051, 0.233])* and competitor collaboration *(estimate* = *0.212, 95% CI* = *[0.037, 0.412])* on firm growth capability mediated by R&D intensity are all significant. However, the indirect effects of vertical collaboration *(estimate* = *0.065, 95% CI* = *[−0.015, 0.191]*), horizontal collaboration *(estimate* = *0.057, 95% CI* = *[−0.031, 0.154])* and competitor collaboration *(estimate* = *0.088, 95% CI* = *[−0.023, 0.228])* on firm growth capability mediated by R&D human capital are not significant. Hypothesis H4 is therefore partially accepted.

**Table 6:** Results of bootstrapping test.

Paths	Estimate	BootSE	95% CI	
			BootLLCI	BootULCI
*Direct Effect (dir):*VC → FGC	0.215	0.137	−0.022	0.521
*Indirect Effect*				
(ind1) VC → RI → FGC	0.123	0.079	0.001	0.305
(ind2) VC → RHC → FGC	0.065	0.054	−0.015	0.191
*Total Effect:* dir+ind1+ind2	0.403	0.178	0.083	0.776
*Direct Effect (dir):* HC → FGC	0.131	0.062	0.008	0.249
*Indirect Effect*				
(ind1) HC → RI → FGC	0.126	0.047	0.051	0.233
(ind2) HC → RHC → FGC	0.057	0.047	−0.031	0.154
*Total Effect:* dir+ind1+ind2	0.314	0.058	0.198	0.430
*Direct Effect (dir):* CC → FGC	0.113	0.172	−0.217	0.464
*Indirect Effect*				
(ind1) CC → RI → FGC	0.212	0.096	0.037	0.412
(ind2) CC → RHC → FGC	0.088	0.065	−0.023	0.228
*Total Effect:* dir+ind1+ind2	0.413	0.187	0.051	0.776

## Conclusion

### Theoretical implications

The theoretical contribution of the study is threefold. First, it provides important advancements to the theory of firm growth from an external perspective. The study examines the relationships between different types of external collaboration and firm growth capability, which expands the research scope of the factors influencing firm growth. The construct of external collaboration in the study, consisting of horizontal, vertical, and competitor collaborations, attempts to go beyond the general concept of collaboration breadth and depth analyzed by Laursen and Salter (2006). It makes the study of open innovation more grounded in practice, as the research question “who are valuable collaborators for an enterprise” is critical to strategic management. This study also enriches the research on the outcomes of open innovation. The consequences of external collaboration have been explored in a large number of domains. Most outcome variables of external collaboration in existing research mainly focused on firm performance (Wang et al., 2015; Zouaghi et al., 2018) and firm innovation (Rodriguez et al., 2017; Brinkerink, 2018; Zhu et al., 2019), while firm growth capability has rarely been involved.

Second, it proposes and empirically tests an integrated model between various types of external collaboration and firm growth capability through the intermediary role of R&D effort. In most extant studies, both external collaboration and R&D effort have been positioned as direct predictors (Zouaghi et al., 2018; Garcia Martinez et al., 2019) or moderators (Ren et al., 2015; Chen et al., 2016; Abdul Basit and Medase, 2019) of firm performance and other outcomes. Little attention has been paid to the potential mediating mechanism. To cover the gap, our study takes R&D efforts as mediators to explore the outside-in influencing mechanism of various types of external collaboration on firm growth capability. The results suggest that the mediating effect of R&D intensity is significant, but the mediating effect of R&D human capital is not significant. It reveals the heterogeneous roles of R&D intensity and human capital in firm growth. Our findings can help researchers to deepen their understanding of the mechanism through which firm growth capability promotes. Moreover, it may enlighten more scholars to develop new studies to explain how firm sustainable growth is achieved under the intense cooperative atmosphere, enriching the literature on sustainability.

Third, the previous research on open innovation mainly centered on firms in developed economies (Chen et al., 2016), and there is a lack of empirical evidence of open innovation strategies in emerging economies. This study contributes to the literature by analyzing how external collaboration affects firm growth capability through R&D efforts in Chinese background. It paves the way for a better understanding of firm growth via external collaboration in the context of emerging economies. We emphasized the particularity in emerging economies, since the firms in different contexts may meet different cultures and limitations in resource, capability, and institution, which may affect the strategies involving collaboration, innovation, and sustainability of those firms.

### Practical implication

It has several important implications for managers. First, enterprises are suggested to strengthen their cooperation with external organizations. Our study shows that vertical collaboration, horizontal collaboration, and competitor collaboration are all beneficial to the advancement of firm growth capability. We recommend firms select suitable partners matching with their internal capabilities in contextual limitations to maintain sustainable growth. Managers are encouraged to take actions for stimulating external collaboration such as building online cooperation platforms and establishing cooperation alliances. Second, enterprises should attach importance to R&D investment, since both R&D intensity and R&D human capital can improve firm growth capability. Managers are also advised to make more effort on R&D activities involving the efficient exploitation of R&D resources. Third, according to the mediation role of R&D intensity, we argue that top managers should take actions to better leverage external collaboration to induce investment in R&D. We trigger the thoughts of diversified functions of external collaboration in the sustainable growth of enterprises.

It also has implications for governments. For example, Chinese central and local governments have introduced many policies involving university-industry cooperation, open innovation, R&D subsidy, talent exchange, and firm sustainability. However, some of them do lack sufficiently theoretical evidence. To some extent, our study could lead governments to make better decisions about firms’ sustainable growth driven by collaborative innovation when they improve their policies.

### Limitations and future research

Despite valuable implications obtained from the results, some limitations do exist. First, cross-sectional data in the study can only reflect the correlations between variables, but cannot infer the causal relationships. The collection of longitudinal data is recommended in the future. Second, the fact that our study only focuses on firms in China limits the generalizability of our results. Future research should therefore conduct cross-cultural analyses to test the robustness of our findings in some other economic entities. Third, self-developed measurement of firm growth capability may be flawed and inadequate, so it needs more tests and improvements. Future empirical research can adopt questionnaire surveys or interviews with executives to capture facets of firm growth capability. Fourth, though the sample size meets the requirement of regression analysis and mediating effect test for parameter estimation, it is insufficient compared with China’s large population and numerous companies. Therefore, we need to expand the sample size in the future. Finally, although our study controls for firm ownership, age, size and industry, we believe that these variables do not cover all the possible contextual differences capable of influencing the relationships examined in our conceptual model. Thus, opportunities for future research should capture other potentially significant control variables, such as firm internationalization and firm hierarchy.

Regardless of the limitations described above, our study brings out some possible future research directions. For instance, we can introduce other variables, such as technology innovation and business model innovation as mediators to further explore the outside-in mechanism of the promotion of firm growth capability. Likewise, scholars may also expand the study by introducing moderating variables, such as market dynamism, technological turbulence, and enterprise culture.

## Supplementary information

Dataset 1

## Data Availability

Some or all data that support the findings of this study are available from the corresponding author upon reasonable request.
